# PBP4: A New Perspective on *Staphylococcus aureus* β-Lactam Resistance

**DOI:** 10.3390/microorganisms6030057

**Published:** 2018-06-22

**Authors:** Thaina M. da Costa, Carolina R. de Oliveira, Henry F. Chambers, Som S. Chatterjee

**Affiliations:** 1 Division of HIV/AIDS, Infectious Diseases and Global Health, Department of Medicine, University of California, San Francisco, San Francisco General Hospital, 1001 Potrero Avenue, Bldg. 30, San Francisco, CA 94110, USA; dacosta.tmiranda@gmail.com (T.M.d.C.); carolinaroberte@gmail.com (C.R.d.O.); Henry.Chambers@ucsf.edu (H.F.C.); 2 Instituto de Microbiologia Paulo de Góes, Universidade Federal do Rio de Janeiro, Rio de Janeiro, RJ 21941-902, Brazil; 3 Instituto de Ciências Biológicas, Universidade de Brasília, Campus Universitário Darcy Ribeiro, Brasília, CEP 70910-900, Brazil

**Keywords:** *Staphylococcus aureus*, β-lactam resistance, Penicillin Binding Protein 4

## Abstract

β-lactam antibiotics are excellent drugs for treatment of staphylococcal infections, due to their superior efficacy and safety compared to other drugs. Effectiveness of β-lactams is severely compromised due to resistance, which is widespread among clinical strains of *Staphylococcus aureus*. β-lactams inhibit bacterial cells by binding to penicillin binding proteins (PBPs), which perform the penultimate steps of bacterial cell wall synthesis. Among PBPs of *S. aureus*, PBP2a has received the most attention for the past several decades due to its preeminent role in conferring both high-level and broad-spectrum resistance to the entire class of β-lactam drugs. Studies on PBP2a have thus unraveled incredible details of its mechanism of action. We have recently identified that an uncanonical, low molecular weight PBP of *S. aureus*, PBP4, can also provide high-level and broad-spectrum resistance to the entire class of β-lactam drugs at a level similar to that of PBP2a. The role of PBP4 has typically been considered not so important for β-lactam resistance of *S. aureus*, and as a result its mode of action remains largely unknown. In this article, we review our current knowledge of PBP4 mediating β-lactam resistance in *S. aureus*.

## 1. Introduction

*Staphylococcus aureus* is an important bacterial pathogen, which causes bloodstream, lower respiratory tract, and skin and soft tissue infections, inflicting high morbidity and mortality worldwide [1,2,3]. Due to its remarkable ability to acquire resistance to antimicrobial agents, a large number of drugs can’ be currently used to treat *S. aureus* infections. β-lactam antibiotics remain as one of the key choices for the treatment of *S. aureus* infections due to their excellent efficacy and safety. However, widespread β-lactam resistance among clinical strains of *S. aureus* limits their use [4].

β-lactams inhibit bacterial cell wall synthesis by binding to Penicillin Binding Proteins (PBPs), which mediate the penultimate steps of bacterial cell wall synthesis [5]. Resistance to β-lactams in *S. aureus* is primarily mediated by two known mechanisms, both of which are horizontally acquired by the bacteria [1]. Resistance to penicillin, the first β-lactam introduced to the health care settings is due to β-lactamase, a secreted enzyme that cleaves the β-lactam ring, inherently present in this group of antibiotics. β-lactamase is encoded by the gene *blaZ*, which is maintained in a plasmid [6]. Resistance mediated by β-lactamase is typically narrow spectrum since it can only inactivate penicillin or drugs that are structurally similar. Currently, a majority of clinical *S. aureus* strains are β-lactamase positive and penicillin is rarely used to treat *S. aureus* infections [2]. Resistance to methicillin, another iconic β-lactam drug that was introduced to the healthcare system in the 1960s, is due to Penicillin Binding Protein 2a (PBP2a; also referred as PBP 2′), encoded by the gene *mecA* or *mecC*, which is maintained in the Staphylococcal Chromosomal Cassette element, SCC*mec* [1,7]. Owing to its remarkable low affinity (due to slow acylation rate) to methicillin, PBP2a allows bacterial cell wall synthesis in presence of high concentrations of the drug, which is otherwise detrimental for the bacteria. Thus, PBP2a provides high-level resistance to methicillin. In addition, resistance mediated by PBP2a is considered to be broad-spectrum since it provides resistance not only to methicillin but to the entire β-lactam class of drug [1,5]. PBP2a is present among all Methicillin Resistant *S. aureus* (MRSA) strains, which, according to a recent estimate, causes >50% of bacteremia caused by *S. aureus* in the USA [8] and is associated with significantly higher health care related costs in the USA [9]. Current treatment of critical *S. aureus* infections can be carried out by ceftaroline, a highly advanced β-lactam drug that received FDA approval in 2010. Several recent clinical surveillance studies however suggest that ceftaroline non-susceptibility in *S. aureus* is on the rise, which is mainly due to PBP2a point mutations [10,11].

Control of both the known β-lactam resistance mechanisms is mediated by similar inducible pathways. When expression of *blaZ* or *mecA* are not warranted, their expression is suppressed by transcriptional repressors, BlaI and MecI respectively. Presence of β-lactam in the bacterial surrounding is sensed by a set of integral membrane proteins, BlaR1 and MecR1 respectively. Subsequently, BlaR1 and MecR1 undergo a site-specific auto-proteolysis, releasing their intracellular zinc metalloprotease domain into the bacterial cytosol. The released zinc metalloprotease degrades BlaI or MecI to de-repress *blaZ*/*mecA* expression leading to resistance [12,13]. Owing to the high similarity of the β-lactam resistance pathways, they are also known to cross regulate each other’s target genes [14,15]. Inducible expression of β-lactam resistance mediators in this manner ensures timely expenditure of cellular resources as warranted.

In addition to PBP2a, which is only present in MRSA isolates, every *S. aureus* strain possess four other intrinsic, core genome encoded PBPs, PBP1 through 4. Currently, the role of PBP1 through 4 in β-lactam resistance or cell wall synthesis and how their expression is regulated, remains elusive to much extent.

## 2. PBPs in *S. aureus* and Their Role in Bacterial Physiology and β-Lactam Resistance

PBPs code for membrane associated proteins that perform the penultimate steps of bacterial cell wall synthesis. They crosslink peptidoglycans, the building block of a bacterial cell wall. β-lactam antibiotics inactivate PBPs by covalently binding to their active site [16]. Since the cell wall is an essential component, inhibition of PBPs through β-lactam drugs compromise cell wall synthesis, which is detrimental to the viability of the bacteria [17,18].

Every *S. aureus* strain possesses four intrinsic, core genome encoded PBPs (PBP 1 through 4), whose molecular masses are 85, 81, 75 and 45 kDa respectively [16,19,20,21,22]. MRSA strains in addition to PBP 1 through 4 also possess the horizontally acquired PBP2a, whose molecular mass is 76 kDa. PBP 1 and 2 play essential role/s in bacterial cell viability whereas PBP2a and PBP4 are non-essential, and as a result genes coding for PBP2a and PBP4 can be deleted from the bacterial genome without any noticeable change in their survival.

Pertaining to bacterial cell wall synthesis, PBPs perform two major functions through their transpeptidase (TPase) and transglycosylase (TGase) activities. Through TPase activity, PBPs crosslink two peptidoglycan units through their penta-glycine cross-bridge, whereas through TGase activity, they crosslink two peptidoglycan units through their sugar units. Although both functions are important for cell wall synthesis, it is the TPase activity of PBPs that confers enhanced cell wall cross-linking and provides structural rigidity to the bacterial cell wall. Every PBP of *S. aureus* possess TPase activity and PBP2 is the only known bi-functional PBP that possesses both TPase and TGase activities [5,23]. For proper cell wall synthesis, both TPase and TGase activities are required and need to be properly coordinated in a spatial and temporal manner, especially during *de-novo* cell wall synthesis.

β-lactams specifically inhibit the TPase activity of PBPs at differing affinities [24]. As mentioned above, PBP2a has low affinity to the entire class of β-lactam antibiotics and is resistant to β-lactam acylation. Thus, in presence of a high concentration of β-lactams that inactivates TPase activity of other PBPs, PBP2a continues to be functionally active due to its low β-lactam affinity and maintains proper cell wall synthesis through its TPase activity [1]. Analysis of the crystal structure of PBP2a suggests that its active site is hidden in a cleft that is inaccessible to the drugs and thus provides a structural basis for its low-affinity to β-lactams [25]. Highly advanced 5th generation cephalosporins, such as ceftaroline, have been designed to access the active site of PBP2a and consequently they are able to efficiently inhibit PBP2a [26]. Besides PBP2a, other PBPs usually play a less prominent role in β-lactam resistance. Clinical strains with point mutations in these PBPs have been previously associated with borderline β-lactam resistance [27,28,29,30] and our recent results as mentioned below have shown that PBP4 can mediate high-level resistance to β-lactams.

In addition to maintaining cell wall synthesis at the cell periphery, PBPs also play a key role in bacterial cell division through mediating cell wall synthesis at the division septum, which ensures proper segregation of cellular materials from a mother to its daughter cells. The division septum is an active site of cell wall synthesis where multiple proteins in addition to PBPs need to structurally and functionally coordinate. The machinery of *S. aureus* that maintains cell wall synthesis at the cell periphery or at the division septum remains poorly characterized. PBP2 (the only bi-functional PBP) has been shown to co-ordinate with PBP2a and PBP4 to mediate cell wall synthesis but their exact mode of action remains unknown [23]. In addition to these, PBP1 has been shown to be important for cell division and septation [31] and PBP3 has been associated with autolysis in *S. aureus* [32].

## 3. PBP4 an Uncanonical PBP in *S. aureus*

PBP4 is roughly half the size of other PBPs and is the only member of Low Molecular Weight (LMW) PBP in *S. aureus*. PBP4 is generally expressed in low amounts compared to other PBPs, as its expression is likely very tightly controlled in *S. aureus* [19,21,33]. Unlike PBP2a, how PBP4 is regulated remains unknown. It is speculated that PBP4 mediated TPase activity is important for generation of highly cross-linked staphylococcal cell wall [23].

In the recent past, elegant work carried out through confocal and epifluorescence microscopy has demonstrated that PBP4 is localized both at the cell periphery and at the division septum. Due to its ability to carry out TPase activity onto partially cross-linked peptidoglycan, PBP4 can repair peptidoglycan defects in cells in a non-*de novo* manner [34,35,36]. Although PBP4 has been shown to play an accessory role in resistance among heterogeneously β-lactam resistant strains of *S. aureus* [37,38], a study carried out in the 1990s depicted PBP4 to mediate moderate level resistance to β-lactam antibiotics [16,39]. We recently identified that PBP4 can mediate high-level and broad-spectrum β-lactam resistance in *S. aureus* that is otherwise known to be mediated by PBP2a in S. aureus [33,40].

## 4. Role of PBP4 in β-Lactam Resistance of *S. aureus*

We recently discovered an uncanonical mode of high-level β-lactam resistance in our laboratory, during experiments carried out to investigate resistance mechanisms to new generation cephalosporins (such as ceftobiprole and ceftaroline) in *S. aureus* [40,41,42,43]. Our results indicate that laboratory passaged strains of *S. aureus* that lacked *mecA* and *blaZ*, the known mediators of β-lactam resistance, can develop high-level resistance to new generation cephalosporins. This indicate that factor/s other than *mecA* and *blaZ* were responsible for this newly discovered resistance phenotype.

Similar passaging studies carried out subsequently with two *S. aureus* strains (COL and SF8300, strains representing an archaic and USA300 backgrounds respectively) in nafcillin (a close relative of methicillin) indicate that this mode of resistance is not only restricted to new generation cephalosporins or to any particular *S. aureus* strain. The passaged strains are resistant to the entire class of β-lactam drugs, which suggests that the underlying basis of resistance is broad spectrum in nature. Furthermore, the high-level resistance that these strains display is comparable to that mediated by PBP2a [33,40,42,43].

To determine the underlying basis of resistance, genome sequencing was carried out, which indicated that mutations associated with *pbp4* were present in each and every resistant passaged strains [40,43]. This suggests that *pbp4* could play a role in β-lactam resistance of these strains. Two different types of mutations associated with *pbp4* were detected: (a) missense mutations in *pbp4* that surrounded the active site serine (S75) of PBP4 [42,43]; and (b) small deletion, duplication and point mutations within the *pbp4* promoter region [42,43]. While the role of the *pbp4* missense mutations is currently unclear, the *pbp4* promoter mutations led to two different phenotypes, PBP4 overproduction and increased cell wall crosslinking [33,40].

A more direct role of PBP4 in mediating high-level β-lactam resistance of these strains came from subsequent molecular genetic studies [40,42,44]. Deletion of *pbp4* and substitution of its active site serine to functionally inert alanine (S75A) turned resistant strains completely susceptible, while complementation of *pbp4* restored resistance, indicating that PBP4 played a quintessential role in resistance of these strains. Further evidence of a pivotal role of PBP4 in this mode of resistance came from subsequent experiments, which showed that *S. aureus* strains that lack both *mecA* and *pbp4* are unable to develop resistance upon similar passaging in the β-lactam drug [42].

To determine the role of each kind of *pbp4* mutation (i.e., missense and promoter mutations associated with *pbp4*) in β-lactam resistance, isogenic mutant strains were created by reconstituting the *pbp4* missense and promoter mutations individually and together in a wild type *S. aureus* strains lacking *mecA* and *blaZ* [44]. Results of resistance assays revealed that *pbp4* missense mutations alone did not play a big role in resistance whereas *pbp4* promoter mutation conferred significant amount of β-lactam resistance when compared to their isogenic wild type strain. Interestingly, *pbp4* missense and promoter mutations together provided a synergistic effect on β-lactam resistance, which indicated that the missense mutations indeed play a role in resistance but how this is achieved is unclear at this point [44].

Clinical significance of PBP4-mediated resistance was unknown until recently. Recent clinical surveillance studies carried out by Argudín et al. detected *pbp4* associated mutations (both missense mutations that targeted its catalytic site and promoter mutations) in clinical strains of *S. aureus* that are either non-susceptible or resistant to β-lactam drugs [27,28]. Interestingly these mutations were detected in both MRSA and MSSA strains suggesting that *pbp4* mediated β-lactam resistance might have been underappreciated clinically in the past and needs to be investigated further.

## 5. Outlook/Future Directions

The role of PBP4 in bacterial cell wall synthesis and β-lactam resistance is currently emerging. Identification of clinically resistant strains bearing similar *pbp4* mutations detected among laboratory-passaged strains underscores its importance and urges further investigation to define the mechanistic basis of this mode of β-lactam resistance in *S. aureus*. Studies carried out thus far have indicated that PBP4 could play an important role in β-lactam resistance through mutations in its gene and promoter. The *pbp4* promoter mutations lead to PBP4 overproduction, which in turn facilitates enhanced crosslinking of the bacterial cell wall. Although it is currently unknown how PBP4 missense mutations mediate β-lactam resistance, our results indicate that they do indeed play a role in resistance [44]. Currently, ongoing crystal structure and biochemical analysis of mutant PBP4 (i.e., PBP4 with missense mutations) will shed more light onto how they mediate resistance in the near future. In addition to this, future studies are needed to determine the regulatory basis of *pbp4* expression, as PBP4 overproduction happens to be an effective way to achieve β-lactam resistance in *S. aureus*. PBP4 being a mono-functional PBP that possesses only TPase activity and since for effective cell wall synthesis, both TPase and TGase activities are required, suggests that PBP4 mediated enhanced crosslinking of bacterial cell wall has to be coordinated with the function of PBP2 (only PBP that possess TGase activity in *S. aureus*).

Previous studies have shown that PBP2 interacts with PBP4 during cell wall synthesis [23]. It will thus be interesting to determine how *pbp4* associated mutations affect the PBP4-PBP2 interaction. It is important to note here that many of our resistant passaged strains as well as the recently identified clinical strains possesses *pbp2* mutations in addition to that in *pbp4*. It is possible that these mutations can promote a more effective PBP4-PBP2 interaction or functional co-ordination. Finally, yet importantly, given the fact that bacterial cell wall is an important component that can be recognized by host cells to elicit an antibacterial response it will be interesting to determine how highly cross-linked cell wall of our resistant strains coordinate a host cell response. Knowledge gained from these studies in the near future will not only enhance our knowledge about this uncanonical mode of β-lactam resistance, but also about the basic biology of how PBP4 functions, its interacting partners, its regulatory basis and how it modulates host cell response.
